# The failure of suicide prevention in primary care: family and GP perspectives – a qualitative study

**DOI:** 10.1186/s12888-017-1508-7

**Published:** 2017-11-21

**Authors:** Gerard Leavey, Sharon Mallon, Janeet Rondon-Sulbaran, Karen Galway, Michael Rosato, Lynette Hughes

**Affiliations:** 1 The Bamford Centre for Mental Health & Wellbeing, Department of Psychology, Ulster University, Cromore Road, Coleraine, BT52 1SA UK; 2 School of Nursing and Midwifery, Queen’s University Belfast, Medical Biology Building, 97 Lisburn Road, Belfast, BT 9 7BL UK; 3 Faculty of Health and Social Care, Horlock Building, Open University, Milton Keynes, MK7 6AA UK

**Keywords:** Suicide, Prevention, Families, Primary care, Psychiatric services

## Abstract

**Background:**

Although Primary care is crucial for suicide prevention, clinicians tend to report completed suicides in their care as non-preventable. We aimed to examine systemic inadequacies in suicide prevention from the perspectives of bereaved family members and GPs.

**Methods:**

Qualitative study of 72 relatives or close friends bereaved by suicide and 19 General Practitioners who have experienced the suicide of patients.

**Results:**

Relatives highlight failures in detecting symptoms and behavioral changes and the inability of GPs to understand the needs of patients and their social contexts. A perceived overreliance on anti-depressant treatment is a major source of criticism by family members. GPs tend to lack confidence in the recognition and management of suicidal patients, and report structural inadequacies in service provision.

**Conclusions:**

Mental health and primary care services must find innovative and ethical ways to involve families in the decision-making process for patients at risk of suicide.

## Background

Most patients who die by suicide, have been in contact with family doctors in the year prior to death [1–3]. Although General Practitioners (GPs) in the UK routinely ask depressed patients screening questions for suicidality [4] many risk factors for suicide are highly prevalent (e.g. depression) and therefore not usefully predictive. Further, impulsivity noted in certain psychiatric disorders and non-diagnosed individuals, has also been linked to suicide and many patients with co-morbid alcohol and substance misuse disorders are difficult to manage [1]. Interventions for the detection, management and outcome of depression and anxiety in primary care indicate positive results for combined strategies of clinician and patient education, nurse case management, shared care with psychiatric services and medication although only with short-term effects [5, 6]. Although various national strategies highlight the importance of primary care in suicide prevention strategies [7, 8] the complexity of recognition and management of suicidal patients by GPs, is seldom acknowledged. Similarly, families are vital in preventing suicide but their experiences in getting and providing care is under-researched. Suicide-bereaved family members have an increased risk for depression, often accompanied by feelings of guilt and blame linked to a quest to understand why the suicide happened [9, 10]. While GPs may be of help to bereaved families, they may also be a source of recrimination when perceived as having failed to prevent it. As part of a multi-perspective programme of research on suicide help-seeking [11] we sought to examine the barriers to effective identification and management of suicidal patients in primary care from the perspectives of GPs and bereaved family members.

## Method

Individual in-depth interviews with (a) relatives bereaved by suicide: and (b) GPs who had experienced the death of at least one patient through suicide.

### Recruitment of sample

**Family participants:** People bereaved by suicide over a 24-month^1^ period were contacted through the Coroners Office Northern Ireland (NI). Seventy-two family members agreed to participate. Following written informed consent, we undertook in-depth interviews with the help of a topic guide that covered the circumstances of the suicide event (e.g. life events, causal attributions), help-seeking and service contacts prior to death. In the latter, we explored difficulties in obtaining appropriate treatment and support within primary care and other services.

**GP Participants**: The GP sample was recruited through the Royal College of General Practitioners (NI). GPs working in Northern Ireland who had experienced the suicide of at least one patient in their care were invited by the College to participate. We were unable to match GPs with the bereaved family sample. Using a narrative approach, we asked GPs to discuss patients who died by suicide, within which we explored (1) contact, recognition and management of suicidal patient; (2) primary care liaison with psychiatric and other services and (3) recommendations for improved care and suicide prevention. We requested that GPs avoid identifying characteristics.

### Analysis

We entered the qualitative data gathered through in-depth interviews with bereaved family members/friends into Atlas-ti 7; a software programme for the management and analysis of text data. The aims of this strand of the study were to obtain a better understanding of help-seeking in relation to the person who died by suicide and then, to examine the impact of suicide on the people who are bereaved by suicide, usually a close family member. Subsidiary areas related to these broad questions included: (a) an understanding of what families believed to have provoked suicide and what might have prevented it; (b) service provision to the deceased and also, the needs and access to services among bereaved families. In undertaking these qualitative interviews, we were not seeking to develop a specific theory, usually associated with grounded theory studies, [12] on help-seeking or the impact on bereaved relatives, but, rather, to offer a detailed, more ‘realist’ picture of relatives’ understanding of the events and experiences leading up to the suicide and a phenomenological grasp of what happens to individuals and their families in the aftermath of suicide. We recognise that this knowledge is always limited and contingent. The participants can only offer a personal view of events, or at times, assisted by the views and knowledge of others, as narrated to them. Nevertheless, the participants must be accepted as ‘close informants’ who knew the people who died by suicide, often intimately. Adopting a realist stance, we have not attempted to ‘interrogate’ their witnessing of the suicide circumstances. Nevertheless, where these emerge, we have sought to show the inconsistencies and paradoxes in the data. Importantly, we have attempted a systematic and comprehensive depiction of the experiences, issues and processes as presented by families without overlaying these accounts with too much intrusive interpretation.

As part of the analytical process, different members of the team examined copies of the first 10 interviews and then met to develop a coding structure, which at first, loosely followed the topic guide in terms of the overall areas of interest and then proceeded to a more detailed thematic analysis of these areas. The codes (and coding frame) were revised at various stages throughout the indexing stage by the team, usually when ambiguities emerged. The 764 codes initially generated from reading and re-reading the interviews were systematized in conceptually clustered matrices [13] as a ‘strategy for conducting thematic analysis to order and synthesise data’ [14]. Wherever possible and without losing all meaning in the transcripts, we have completely removed all identifying characteristics of the people who died and the participants who provided these accounts. The data are mainly presented in a thematic analysis and therefore may be prone to a degree of fragmentation or disconnection between beliefs, attitudes, events and experiences, in which the ‘full narratives’ appear to get lost.

## Results

### Sample

**Close informants:** 72 women and men closely connected to the person who died. The participants lived in a spread of urban and rural areas. The informants age-bands and relationship to the deceased are given in Table 1. Sociodemographic details of the deceased have not been published to secure anonymity.

**Table 1:** Age and Kinship of Participants

Kinship	Age						
	16 to 34 years	35 to 44 years	45 to 54 years	55 to 64 years	65 plus years	Age missing	Total
Brother	1	3	3				7
Daughter	1	2					3
Father	1		1	3		1	6
Husband			2	1	1		4
Mother		3	2	4	1	1	11
Sister		2	4	3	1	1	11
Son	2		1		1		4
Wife	1	2	7	3	2	4	19
Other/Friends		3		2	1	1	7
Total	6	15	20	16	7	8	72

**General Practitioners:** 11 women and 8 men, from a diverse range of primary care settings, (socio-economic and urban, semi-urban and rural populations). The participants were all long-serving practitioners, between 15 and 30 years’ experience. Each had been involved in the care of more than 2 people who died by suicide, with several GPs indicating more than 10 cases.^2^

### Family perspectives on primary care

In the first section, we highlight the positive aspects of primary care. The main section covers the barriers to effective care and suicide prevention. The themes relate to recognition, management and poor continuity of care (Table 2).

**Table 2:** Barriers to recognition, effective management and suicide prevention

GP (MIS) RECOGNITION	
PATIENTS RECOGNITION	
No prior history of mental illness	
Stigma and denial of mental illness	
Limited contact	
Somatic presentations	
Substance misuse	
GP FACTORS	SUICIDE ASSESSMENT/SCREENING
Limited competence (mental health	Regarded as simplistic and intrusive
Low confidence	Limited time for psychological care
Negative views of mental health patients Scepticism of patient suicidal intent	Barrier to therapeutic engagement
	Undermines patient trust
Belief in patient stoicism (stereotyping)	
GP MANAGEMENT (PATIENT FACTORS)	GP MANAGEMENT (psychiatry liaison)
Rejection of diagnosis	Long waiting lists
Medication non-adherence	Psychiatry focus on psychotic illness
Substance misuse	Inadequate follow-up
Multiple contacts	Functional split model
	Fragmentation of services
	Loss of shared knowledge and expertise

#### Compassionate care

Relatives were satisfied with GPs who showed a humane as well as a professional interest in the suicidal patient, maintained contact, discussed treatment options, were flexible and communicated with relatives. They also made domiciliary visits if requested.

Consultations with the same GP reassured relatives that ‘nothing was being missed’, which, in turn, patients’ and families’ trust. Such GPs were perceived to be more aware of the patient’s and family needs, and their specific contexts and environments. They were considered to ‘have done their best’ to prevent the suicide:

The GP was quite proactive. He had a good relationship with the GP and actually spoke at length with the GP, yeah, because he had the same GP throughout and he would have spoken quite highly of his GP (P71, Wife).

She really thought a lot of him [the GP]. He was actually the GP that came down the night that she had to go to the hospital to get her stomach pumped and he knew the build up to it and knew her circumstances and knew everything. … Possibly she had told Dr. [name of doctor] the whole story and he knew what was happening. So, he maybe tried to keep the husband at a distance (P15, Sister).

In this last quote, we get an indication of the fine balance that GPs are obliged to preserve; keeping family members involved (some of whom may have contributed to the patient’s problems) while securing patient confidentiality.

### GP recognition

#### Patient concealment

Participants, generally, acknowledged that the GP’s assessment of risk is grounded in the patient’s communication of emotions and intentions. In many cases, families described their relative’s refusal to ‘open up’. Consequently, some families accompanied the patient to the surgery but were then thwarted by the patient’s denials of depression or suicidality. These ‘contests’, between the patient and other family members sometimes provoked inter-family frustration and resentment. Various participants reported difficulties in discussing depression with GPs or how doctors misinterpret, or possibly ignore, patient communication. In other cases, GPs failed to recognise the significance of an unusual visit by a depressed patient. Failure of GP suicide risk assessment was common among patients living with chronic pain. In the following quote, a daughter attributes her father’s suicide to unmanaged arthritic pain:

I think it was only about two weeks before he died he had to go back to the doctor for his pain relief and he really needed them upped but the doctor gave him his usual prescription, and he had mentioned being quite down about it all. The doctor had … because my mum always went with him, and the doctor had said, ‘*Well, are you so depressed (that you might) harm yourself?*’ And my daddy just shrugged his shoulders. But then the doctor said, ‘*Well, there's your pain relief and make another appointment and we’ll deal with your depression*.’ So, to me, if somebody shrugs that should be enough. That doesn’t mean, ‘*No, I’m fine.*’ (P12, Daughter).

Others asserted that GPs missed the signs of depression expressed in frequent contact with the surgery.

To me, the doctor should have noticed it. She was in the doctor’s maybe twice a day. […] She was neglected, pure neglect, wasn’t given the appropriate treatment or assessed right or anything, and I do blame the GP. (P49, Sister).

#### Brief consultations: “The ten-minute rule”

In many narratives, families complained that GPs missed suicidality because of a busy appointments schedule, an argument that recurs in the GP interviews. Limited time was thus regarded as a key barrier to securing patient trust, communication about mental illness and suicide risk assessment:

Doctors are ten minutes and you’re out and it’s not easy for anybody to go, there's always the stigma of mental health problems … (P37, Father).

#### Antidepressants and limited treatment options

Anti-depressant medication and limited alternative treatment options, was a major source of dissatisfaction. In several cases, patients stopped taking medication because they feared addiction or experienced side-effects, e.g. lethargy, erectile dysfunction. The term ‘*reluctant*’ was commonly used, often expressed as ‘common knowledge’ by the suicidal person that antidepressants are ineffective, or took too long to ‘work’.

He didn’t like taking them so he stopped taking them for whatever reason. He should have been taking them maybe. There’s a lot of people don't like taking them because they think, ‘*I’m hooked on these things now forever*’ (P8, Friend).

In other cases, patients rejected medication because it conflicted with their explanatory model of the problem. For example, as reported by a family member, one patient with post-natal depression felt that her problems were related to her “marriage and no matter how much medication she took it wasn’t going to help her”. She was given anti-depressants but didn’t take them.

A recurrent complaint was that GPs were over-reliant on medication and took little account of patient reluctance and family concerns

I wanted an appointment to see the doctor and asked the receptionist, I said, ‘*I’m here about* my husband. *Could you* give me an appointment?’ ‘*No need*,’ she said ‘*at all, there’s another prescription*.’ […] My husband was given different types of … sleeping tablets, tranquilizers. I said, ‘*What do I do with the rest of them? He’ll not take them; that is why I want to see the doctor. He thinks I’m drugging him*.’ […] I was at the end of my tether. (P58, Wife).

#### Failure to review

A lack of effective monitoring added to the perceptions by relatives that patients were ‘*being fed*’ medication, as one person expressed it. The family’s inability to monitor medication is also noted.

The only treatment … he was on anti-depressants; […] and what we didn’t know, he had opted to stop taking them without medical advice. How long he had stopped taking them, I’m not aware even at this stage. I presume it would probably … it could be months, it could be a year, I don't know. But he had elected himself to stop taking them (P19, Brother).

### General practitioners perspectives

The key thematic areas identified by GPs in describing barriers to suicide prevention are as follows: (1) Recognition and management of suicidal people; (2) Liaison and communication with Mental Health Services; (3) Dealing with bereaved families; (4) Professional and personal impact of patient suicide.

### Recognition and management

#### No contact

GPs noted that suicides on their register, predominantly men, had seldom or never made any contact. Others, known to the GP as having a history of psychiatric problems, had quit the area and didn’t re-register elsewhere. When patients don’t contact or attend appointments, the default assumption among GPs is that they are well.

#### Stigma

GPs substantiated the relatives’ narratives as to the deterrent strength of stigma about seeking help for mental illness; especially in their accounts of suicide among men from small and rural communities where for cultural and professional reasons, the shame of mental illness is profound.

Out in farming communities in particular, people are very … they pride themselves in being very robust, and men in particular don't seek help unless it’s something quite seriously wrong with them, be it on a physical level, and often you don't see people who are farmers with mental problems unless they are brought in by other relatives. … It would be seen as unmanly (…) if you have to come in and tell the doctor that you’re depressed or you’re not coping or whatever. (GP1)

#### Assessing risk

GPs acknowledged the lack training or confidence in dealing with psychiatric disorders. With severe and enduring mental illness, psychosis for example, GPs felt safer referring to secondary care. With common mental disorders and suicidality, recognition and management is unclear. Thus, many of the GPs in deprived, inner-city areas contend with problems of drug abuse and gang violence. Some report feeling overwhelmed by the demand for sick notes and psychiatric medication, according to some, often illegitimately. One GP described being confronted by patients who “demanded diazepam or they would kill themselves.”

A lot of people sick, mentally ill, and a lot of people pretending to be mentally ill to get disability living allowance. (GP9)

#### GP scepticism

It was commonly argued that general practice is currently dominated by a concern for suicide rather than mental illness. One view is that many people who contact the GP stating suicidality, are unlikely to be so. The suicidal declaration is regarded as a recently emerging form of presentation, in which the patient, because of current adverse events or circumstances, is finding life very difficult; interpreted by some, as a strategy to rouse medical care or secure disability allowance. In the next quote, the GP indicates something of a cultural response to personal problems.

I suppose the problem is that the people who come in saying they’re suicidal are then, I suppose, taking up work … they take up quite a considerable amount of work, and there are one particular family going through here at the minute who have had three or four people from the same family, girls perhaps one or two years older than each other, presenting with the same suicidal ideation over the last few weeks, which takes up a considerable amount of time for CPNs. So the lag then that you see is people who perhaps aren’t perceived as being suicidal but have acute mental health problems that probably need dealing with but the system’s stretched trying to deal with people that are presenting with these crises situations. (GP14)

Nevertheless, we also noted that although GPs suggest a degree of awareness that the patient may have other anxieties, these remain unexplored.

#### Risk assessment process

GPs are now required to screen for potential suicidality using standard questions, which, commonly, are considered to be counterproductive and provoke defensiveness among patients. One GP mentioned patients “bristling” or getting upset; asking, “Do you think I’m mad!”. Typically, GPs find the suicide protocol as a barrier to therapeutic engagement or more suited to GPs who are not confident, commenting that they find “more sensitive” ways of determining suicidal thoughts among patients.

You have to ask them about their alcohol and their social history and their debt and their family support, and stuff you did anyway but never actually ticked boxes, especially if you actually print it out and sit in front of the patient and do it, that totally depersonalises somebody, because “why does the doctor want to know how much I earn or whether I’ve got any credit card debt?” (GP3)

#### The ten-minute rule

“I think that we need more time with patients. The demand is the problem, accident and emergency is on its knees. It is not a problem about GPs, it is a problem of impossible demand.”

As noted previously, the stigma of mental illness and patient reticence to discuss ‘personal’ problems often inhibits any meaningful intervention by GPs. Patients often require gentle encouragement to disclose their troubles. The issue of limited time was universally noted as a barrier to the recognition and management of any underlying mental health or emotional problems.

Personally, I … if somebody needs to talk, they need to talk and the waiting time goes out the window, and my patients tend to know that and my reception staff are very good at saying ‘she’s running a bit behind today’. (GP5)

Few GPs were as relaxed about the 10-min rule as the GP above. Patients often begin to discuss the ‘true’ presenting problem as their 10 min come to an end, leaving the GP with difficult choices to make. In the following quote, the participant relates that counselling, an important GP skill is being eroded by work overload and bureaucratisation. From this perspective, GPs decreasingly provide the humane and compassionate aspects of primary care.

My job is changing, that little bit of counselling I do as I go through my job, there’s not time for it anymore. I have to pass that onto somebody else. It’s a bit of a struggle to state some of the things I do well, there's not time for this anymore; that's becoming someone’s else’s job, whether my patients want it to be or not or whether I want it to be or not. I can't keep doing my job and stay sane and do all of this anymore because there's just not time. (GP8)

#### Suicide as unpredictable

He wasn't on drugs, he didn't drink, he had friends, he hadn’t had a relationship bust up; there was nothing that you could point other than his cousin did it. (GP5)

The unpredictability of suicide was a repeated issue. In the above quote, the GP reflects that this young man’s suicide was emulative. GPs remarked on the heterogeneity of the suicide, in terms of the patient characteristics, social contexts and presenting problems. The unpredictability may arise in various ways. For example, through *somatic presentations* or when patients may have attended surgery for some time but show no indication that they may be depressed or suicidal. Such consultations are often for physical problems, common among older patients, some of who have been managing chronic, complex conditions. Such patients are highly regarded for their long-term stoicism, presenting a ‘cheery front’ and then leaving doctors “quite taken aback” when suicide occurs.

No, he never ever presented with any psychiatric illness. Even in the bad times when there were assassinations going on and all sorts of things where he really was at risk, he never sought any help with anxiety, depression, nothing. (…) So I knew him reasonably well and he didn’t use drugs, he took a drink but not to excess. He was not predictable; I could never have spotted him for suicide, no way. (GP3)

In other cases, the presenting problems seemed minor or non-existent.

The day I saw him, for example, he was more concerned about how he looked rather than basically … and I sort of said what is actually bothering you and he said the spots on his face. He didn't sort of have any … it didn't seem to be a body image problem and we did explore other sort of symptoms. I did ask about sleep, appetite and his general enjoyment of things, and he certainly didn’t seem unkempt or anything like that. He seemed a typical sort of young person. (GP12)

### Paradoxical presentation

Patients who have been treated for depression may complete suicide in the midst of ostensibly positive signs and outcomes. Thus, they appear to be responding well to treatment, returning to work, engaging with community. In the following case, the patient met the GP and reported that her “life was on-track”; she had a new job and a new house.

She said, “Doctor, everything is fine…and then, two days later, the police walked in; she had been on the booze and hanged herself. What do you do about that?”

### Continuity of care and poor engagement

In various narratives, suicides happen despite an apparent high level of attention by services, both statutory and voluntary. In one case, a young man had a difficult existence that included family breakup, a previous suicide attempt aged 14 years, unemployment and substance misuse. His history of poor engagement with mental health services echoes other interviews.

Two or three months prior to his suicide he consulted with a couple of the different doctors and we changed his anti-depressants and we’d brought him back for review. He didn't keep the review dates and when he did come in it was about some other health problem. He denied any self-harm and he denied any sort of suicidal tendencies. He did display some anger management problems, which we’d referred him to men-to -men project and also to a counsellor. He had been seen also by (a voluntary sector organization) and then prior to his committing suicide he actually had been seen in A&E but we didn’t get the letter until after his demise. So, he’d actually been seen by Crisis Response Team. (GP12)

The problem of poor engagement noted by this GP was balanced against the issue of continuity of care. She reported that the patient was somewhat manipulative and had deliberately sought the assistance of different GPs and had been offered a range of support. In other cases, continuity of care was influenced by family behavior, often driven by stigma. For example, in one case, the GP reported that the family had “protected” their son for a considerable time before seeking treatment. When they did so, they also tried to obtain care from private healthcare (described as a “disaster”). Moreover, the family neglected to convey to the GP, various indicators of suicidal intent; revealed only after his death.

### Alcohol and drug use

GPs describe the lives of various suicide patients as chaotic and in which alcohol plays a significant role, either as self-medication when depression hits or as a precursor factor in the loss of job, family and contact with the criminal justice system. The presence of alcohol is regarded as obscuring diagnosis and hindering treatment.

There’s been a few who are almost regulars where you just don’t know are they really suicidal or is it a cry for help, and there's been a few repeated ones would take overdoses who we’re all aware of, or maybe the odd cut wrist and stuff, but more tentative wounds. But mostly overdoses, quite often tied in with drugs, or alcohol particularly (GP15)

### Psychiatric services

GPs’ relationships with mental health services were variable, shifting from complementary and highly satisfied to the fractious and disturbing. The variance was partly determined by locale and the structural organization of psychiatric services. Although some GPs had good relationships with local mental health services, particularly crisis intervention and home treatment teams, they described substantial gaps within the community care of people with severe depressive disorders. Additionally, acknowledging that many suicides appear unpreventable, they point to instances when suicide should not have been the outcome. In brief however, the dominant issues documented by GPs regarding mental health services relate to the following: (1) Perceived inadequate assessment; (2) Delays to hospitalization; (3) Inadequate or absent follow-up in the community; (4) Lack of communication with primary care; (5) Absence of shared knowledge and expertise..^3^

### Inadequate response

There was a strong perception that psychiatrists are sceptical of the GP’s professional judgment or at least, a tendency to minimize the GP’s assessment of the urgency of the problem. One GP felt that the structure of a ‘One-point referral’ system was unhelpful. In other instances, GPs believed that patients were sketchily assessed and then discharged with minimal safeguarding. In the following case, the GP felt that sufficient warning signs had been raised but that the assessment arrangement had been “dragging on for days”. She argued with psychiatry that she knew the patient very well, much better than mental health services.

I was very upset because I felt that if he had been sectioned, he would have been alive and we could have worked through the issues he had. (GP1)

Providing a psychiatric assessment via the telephone was problematic when the patient was present in the surgery but wanting to leave. In some cases, the GPs could not convince the clinician on the other end of the phone that the patient required admission. In other cases, differing clinical judgments are made about the patient’s mental state and the assessment of risk. In one case, a female patient experienced an alarming personality change. Just over 65 years, the patient fell between adult mental health and old age psychiatry. Clinicians disagreed as to her mental state and capacity. The patient took her own life in the midst of clinical wrangling. Other suicides may happen because psychiatric services were stretched too thinly.

After his discharge from hospital into the care of the home treatment team, he was due to have daily reviews (…). The day after his discharge, they (home treatment team) reviewed by home and decided with him that they didn’t need to see him the next day. So, the first contact with (services) was to downgrade his review and the next day he killed himself. (GP13)

The same GP argued that psychiatric services tend to neglect people who are depressed, concentrating on severe and enduring disorders instead. In several cases, concerned about their deterioration, he referred patients with a severe depression to psychiatric services, only to be told, “we don’t think this is our problem, could you send the patient to some of the voluntary services”. He stated that it was not reasonable for a GP to undertake outreach for people with severe depression and who may be suicidal.

### Communication and liaison with psychiatry

GPs complained of attempting, desperately on many occasions, to convince the psychiatric gatekeepers of the need for an emergency referral.^4^ In other instances, some patients were described as being at increased risk because of transitional boundaries. For example, young people who having been seen in Child and Adolescent Mental Health Services (CAMHS) but were not transferred into adult care. When connections and relationships dissolved in these structures, so too did the continuity of care and some GPs felt that some people might still be alive today had these barriers to communication and referral not existed.

The organisation of mental health services in some Health & Social Care Trusts had undergone considerable change leading to a fragmentation of psychiatric services into different specialist sectors (for example, recovery, addiction, emergency).

They would overlap and you’d have the difficulties of getting the right person; people would get lost between the cracks. But the actual individual, when you got them, was super. I felt that there was a loss of continuity. The GP is the only person, really, that had any picture of continuity. (GP6)

Thus, the functional split within psychiatric services removed a valued, direct and personal connection between primary care and psychiatrists, through which advice or concern about a patient’s mental state could be quickly resolved by a phone call. Once removed, GPs felt that mental health services may not be operating in the best interests of the GP or the patient. The suspicion that mental health services are “off-loading” was noted elsewhere. There is some concern that the voluntary sector is expected to fill in the gaps but this is becomes part of a ‘revolving door’ scenario. There were various views about the value of voluntary sector provision of psychological therapies but most found it hard to comment, simply because communication between primary care and the sector was minimal with no feedback about patients referred. The disjunction between primary care and mental health services had deeper implications and GPs wanted the development of closer liaison and knowledge sharing.

Greater co-operation between the services, at present I have no contact with the psychiatric services at all. There is no personal contact, it would really help if we had more regular meeting or case discussions with them that would be a learning opportunity for us; we might be able to fill them in on some of the pressures.

Probably a greater appreciation for each other’s roles and what we can do. (GP14)

## Discussion

Contact with primary care services prior to suicide is common, leaving opportunities for intervention [3, 15]. In previous work from this study we found that most primary care contacts were related to mental health problems, increasing over time [16]. The likelihood of contact was increased among women, older people and among people with substance misuse problems and common mental disorders [16]. The prevention of suicide often depends on a delicate collaboration between multiple agents and agencies in which availability and timing, knowledge and communication, relationships and trust, all contribute. Certainly, prevention is not clear cut [17] In common with other research in this area we found considerable and complex challenges to engagement with the health care system, some structural, others cultural [18, 19].

In this paper we sought to explore these relationships from the perspectives of families and general practitioners. Families, while not universally reproachful of health services, raise important concerns about the recognition and management of suicidal risk in patients. Other evidence from the UK indicates that a high proportion of patients take their own lives within a month of discharge from services, suggesting a poor follow-up and engagement with the families into whose care they are often left [20]. The brevity of the GP consultation was regarded as the foremost barrier to recognition and treatment of mental illness. Bearing in mind that the stigma of mental illness in many cases had consistently delayed any contact with services, families were especially distraught when, despite their efforts to get help, GP’s appeared to accept the patient’s misleading presentation of the problem. In such cases, suicides sometimes occurred shortly after contact or in the midst of being treated for other chronic, physical health conditions.

General practitioners provided an alternative but sometimes, complimentary perspective, to that of the family participants. Theirs was a complicated and uncertain position in suicide prevention in primary care, as it was regarded commonly as unpredictable. Thus, even when individuals are diagnosed and treated appropriately, suicide still happens. Nevertheless, in line with evidence from the USA and elsewhere, [21]. GPs also acknowledged, that some suicides may have been preventable, ‘failures’ regarded either as limited personal competency or a systemic failure, the result of poor liaison and decision-making. We found widespread agreement that GPs are ill prepared and ill equipped in the management of mental illness, compounded by the pressure of patient waiting rooms and waiting lists [22]. In views that chimed with those of relatives, high levels of demand ensures that people with complex psycho-social needs are afforded only superficial opportunity to articulate their problems. Given that some GPs feel that ‘pro-forma’ questions about an individual’s suicidal ideas and behaviours undermine patient trust and engagement, there is a need for research on how best to support GPs in screening for suicidality in ways that don’t compromise patient trust.

The pressure of time and low confidence in mental health care, appears to push GPs into an overreliance on pharmacological treatment, leaving little space for patient preference or extended discussions with patients about the benefits of such treatments. While, some GPs presented the notion of patient-GP partnership, a psychological contract with obligations and responsibilities on both sides, the dominant approach, albeit for pragmatic reasons, appears to be, ‘take it or leave it’. As noted in the participant interviews, many of those who died by suicide were dissatisfied with their medication and simply stopped using it. This and many other issues raised by families, run contrary to current NHS clinical guidelines [23] for patient-centred care, communication and self-management. Moreover, evidence suggests that outpatient contact, rather than hospitalisation, and psychotherapy or other brief psychological treatments may be effective for patients following a suicide attempt or high risk of repeat self-injury [24].

Systemic failures refer predominantly to communication breakdown between primary care and psychiatry. These arise from the split between the traditional approach, that is, a sectorised model and the functional split model [25]. In the former, a single consultant provides both hospital and community care within a designated geographical catchment area. In the functional split model, various other specialist teams are established such as recovery, crisis resolution and home treatment teams. Where this model has been adopted, consultant psychiatrists are no longer responsible for patients across the range of treatment settings. For many GPs, this lack of a central contact is a challenge to GP care of people with mental illness, on a range of levels and issues. Social capital in the form of knowledge-sharing, advice and trust-building, is quick to disappear.

## Conclusions

Our study provides first-hand accounts of the difficulties of managing suicidal patients within primary care services. While some completed suicide events can appear spontaneous or impulsive, suggesting that they were unavoidable, there may be considerable failures to detect individuals at risk. Fragmentation of care is poor and inefficient care. The costs to society and families are considerable. We know that GPs lack training in mental disorders and therefore it is imperative that we find innovative ways of integrating mental health services within primary care settings, perhaps funding for mental health nurse specialists within practice or regular mental health clinics led by psychiatric teams. The failure of services to prevent suicide is heightened by an inability to engage families in a much more constructive and helpful partnership. Communication and liaison between psychiatric services, primary care and families requires better is currently poor and E-technology has the potential to monitor change and deterioration in mental states and to rapidly transfer these data easily between caregivers and services. Lastly, too often psychiatric and medical services deny families the knowledge they need to provide support and care; they do so on the grounds of patient confidentiality. While this is sacrosanct, these can be set aside where a life is at risk. Physicians can and should be better informed on these issues.

## Abbreviation

GP: General Practitioner

## Footnotes

1 1st March 2007-28th February 2009

2 Within the interviews, three doctors acknowledged a personal connection with suicides outside their professional role. These deaths are likely to have influenced such doctors’ views on suicide and their professional engagement with suicidal people.

3 Other, perhaps more minor points that were mentioned in the interviews relate to concerns about the long waiting lists and the refusal of mental health assessment teams to see people who are intoxicated or have taken drugs.

4 The issue of alcohol use by patients was mentioned as a barrier to emergency referral. Again, given the association between alcohol use and suicidal behaviour, the rejection of such people by emergency services seems to be contrary to suicide prevention.

## References

[1] NCISH (2014). National Confidential Inquiry into suicide and homicide by people with mental illness: suicide in primary Care in England: 2002–2011.

[2] Pearson A, Saini P, Da Cruz D, Miles C, While D, Swinson N, Williams A, Shaw J, Appleby L, Kapur N (2009). Primary care contact prior to suicide in individuals with mental illness. Br J Gen Pract.

[3] Luoma JB, Martin CE, Pearson JL (2002). Contact with mental health and primary care providers before suicide: a review of the evidence. Am J Psychiatr.

[4] Gilbody S, Sheldon T, Wessely S (2006). Should we screen for depression?. Br Med J.

[5] NHS centre for Reviews and Dissemination (2002). Improving the recognition and management of depression in primary care. Eff Health Care York Royal Soc Med Press.

[6] Heideman J (2005). Interventions to improve management of anxiety disorders in general practice: a systematic review. Br J Gen Pract.

[7] O'Connor E (2013). Screening for and treatment of suicide risk relevant to primary care: a systematic review for the U.S. preventive services task force. Ann Intern Med.

[8] Mann J, Apter A, Bertolote J (2005). Suicide prevention strategies: a systematic review. JAMA.

[9] Hawton K, Simkin S (2003). Helping people bereaved by suicide: their needs may require special attention. Br Med J.

[10] Cerel J, Roberts T (2005). Suicidal behaviour in the family and adolescent risk behaviour. J Adolesc Health.

[11] Mallon, S., et al., Suicide and Life-Threatening Behavior. Patterns of presentation for attempted suicide: analysis of a cohort of individuals who subsequently died by suicide, in press. 10.1111/sltb.12134 25346168

[12] Glaser B (1992). Basics of grounded theory analysis.

[13] Miles, M.B. and A.M. Huberman, Qualitative data analysis: an expanded sourcebook. 2nd ed. 1994, Thousand Oaks CA: Sage. 338.

[14] Pope C, Mays N (2008). Qualitative research in health care.

[15] Ahmedani BK (2014). Health care contacts in the year before suicide death. J Gen Intern Med.

[16] Leavey G (2016). Patterns and predictors of help seeking contacts with health services and general practice detection of suicidality. BMC Psychiatry.

[17] O’Connor E (2013). Screening for and treatment of suicide risk relevant to primary care: a systematic review for the U.S. preventive services task force. Ann Int Med.

[18] Graham RD, Rudd MD, Bryan CJ (2011). Primary care providers’ views regarding assessing and treating suicidal patients. Suicide Life Threat Behav.

[19] Vannoy SD (2011). Now what should I do? Primary care physicians’ responses to older adults expressing thoughts of suicide. J Gen Int Med.

[20] NCISH (2014). National confidential inquiry into suicide and homicide by people with mental illness: suicide in primary care in England: 2002–2011.

[21] Feldman MD (2007). Let’s not talk about it: suicide inquiry in primary care. Ann Fam Med.

[22] Pollock K, Grime J (2002). Patients’ perceptions of entitlement to time in general practice consultations for depression: qualitative study. Br Med J.

[23] National Institute for Health and Clinical Excellence, Patient experience in adult NHS services: improving the experience of care for people using adult NHS services. London; 2012. 34260162

[24] Comtois KA, Linehan AA (2006). Psychosocial treatments of suicidal behaviors: a practice-friendly review. J Clin Psychol.

[25] Laugharne R, Pant M (2012). Sector and functional models of consultant care: in-patient satisfaction with psychiatrists. Psychiatrist.

